# Innovations in Early Lung Cancer Detection: Tracing the Evolution and Advancements in Screening

**DOI:** 10.3390/jcm13164911

**Published:** 2024-08-20

**Authors:** Lindsey B. Cotton, Peter B. Bach, Chris Cisar, Caitlin A. Schonewolf, Demetria Tennefoss, Anil Vachani, Lisa Carter-Bawa, Ali H. Zaidi

**Affiliations:** 1 DELFI Diagnostics, Inc., Baltimore, MD 21224, USA; 2 Pulmonary, Allergy, and Critical Care Division, University of Pennsylvania Perelman School of Medicine, Philadelphia, PA 19104, USA; 3 Center for Discovery & Innovation at Hackensack Meridian Health, Cancer Prevention Precision Control Institute, Nutley, NJ 07110, USA; 4 Allegheny Health Network Cancer Institute, Pittsburgh, PA 15224, USA; ali.zaidi@ahn.org

**Keywords:** USPSTF, lung cancer screening, low-dose computed tomography, blood-based testing, NSCLC, screening adherence, cell-free DNA, liquid biopsy, fragmentomics, DELFI

## Abstract

Lung cancer mortality rates, particularly non-small cell lung cancer (NSCLC), continue to present a significant global health challenge, and the adoption of lung cancer screening remains limited, often influenced by inequities in access to healthcare. Despite clinical evidence demonstrating the efficacy of annual screening with low-dose computed tomography (LDCT) and recommendations from medical organizations including the U.S. Preventive Services Task Force (USPSTF), the national lung cancer screening uptake remains around 5% among eligible individuals. Advancements in the clinical management of NSCLC have recently become more personalized with the implementation of blood-based biomarker testing. Extensive research into tumor-derived cell-free DNA (cfDNA) through fragmentation offers a novel method for improving early lung cancer detection. This review assesses the screening landscape, explores obstacles to lung cancer screening, and discusses how a plasma whole genome fragmentome test (pWGFrag-Lung) can improve lung cancer screening participation and adherence.

## 1. Introduction

Globally, lung cancer is the number one contributor to cancer-related mortality regardless of gender or ethnicity and it accounts for approximately 2.2 million new cases and 1.8 million deaths each year [1,2]. The 5-year relative survival rate for late-stage (stage IV) diagnosis in the United States is 4% [3]. Yet, for early-stage cancer (stage I), the 5-year relative survival rate increases to 55% [3]. Despite known benefits of lung cancer screening with low-dose computed tomography (LDCT) supported by extensive randomized clinical trial data derived primarily from the National Lung Cancer Screening Trial (NLST) [4] and Dutch-Belgian Randomized Lung Cancer Screening Trial (NELSON) [5], most lung cancer cases are still detected at a late-stage, and LDCT uptake in the US remains around 5% [4]. Each year, more people die of lung cancer than of breast, cervical, and colon cancers combined [3]. Other cancers with established screening methods exhibit annual screening rates ranging from 67% to 76%, which amounts to screening rates 13 to 15 times higher than that for lung cancer [6]. While several factors contribute to poor uptake of lung cancer screening, eligibility criteria and stigma around smoking have had a significant impact over time. Compared to other cancers with available screening modalities, eligibility for lung cancer screening requires a history of smoking, whereas screening for breast, cervical, and colon cancer is only contingent upon age. Decades of shame-based campaigns have caused many individuals who smoke to omit their smoking status to providers. Individuals diagnosed with lung cancer report greater levels of stigma and resultant psychological distress in comparison to those with other cancer types [7]. Consequently, this historical pattern has compounding effects, as the population eligible for lung cancer screening faces a significantly higher disease risk but often experiences delayed access to medical interventions that are empirically proven to save lives. Lung cancer deaths were nearly 3-times as high as breast, cervical, and colon cancers despite LDCT of the chest being effective and efficient; about 323 individuals need to be screened to prevent one lung cancer-related death compared to 543, 1140, and 588 for breast, cervical, and colon respectively [8,9,10].

Primary care providers play a vital role in spearheading public health awareness and driving a paradigm shift concerning the perceived stigma surrounding lung cancer. In the US, access to lung cancer screening is contingent upon a referral from a healthcare provider. Findings from studies of populations eligible for lung cancer screening suggest that a substantial number of participants expressed a willingness to undergo screening if their primary care provider recommended it [11,12]. This underscores the pivotal role of healthcare providers in shared decision-making and the importance of providers being educated on resources that increase patient access to screening opportunities.

One such opportunity includes liquid biopsy tests, which have emerged as a promising development in cancer detection. Liquid biopsy tests offer a convenient and more accessible testing approach by analyzing biomarkers in a blood sample to assess the presence of cancer DNA. Results would be evaluated further by imaging, thereby improving screening uptake and adherence. Such developments represent a significant advancement in the pursuit of early detection that could ultimately lead to improved health outcomes. While there are several targets for liquid biopsy (i.e., genomic markers, methylation profiles, circulating tumor DNA), the purpose of this review is to detail the challenges of current LDCT based screening, explore the development of blood-based DNA testing in the field of lung cancer screening, specifically whole genome fragmentome based testing which is currently commercially available, and then briefly discuss how blood based testing could supplement LDCT.

## 2. The Evolving Landscape of Lung Cancer Screening

### 2.1. The Value of Imaging to Detect Cancer Early

Associations between the incidence of smoking and lung cancer were first identified in the 1950s, marking a turning point in understanding risk factors and initiating a quest for effective screening methods [13]. By 1970, the first randomized controlled trial was launched in the US using chest radiography (CXR), the standard method for chest imaging in that era [13].

The development of computed tomography (CT) scanners and its use on patients in the early 1970s shifted medical conversations around imaging. Several single-arm observational studies using LDCTs as a screening tool were conducted in the 1990s, and by 2000, the National Cancer Institute (NCI) initiated a feasibility study that randomized high-risk individuals with a smoking history to CXR or LDCT of the chest. This feasibility study led to the NLST, a multi-center randomized controlled trial with 53,454 participants that demonstrated a 20% reduction in lung cancer mortality and a 6.7% reduction in overall mortality using LDCT compared to CXR [4]. An extended analysis in 2019 confirmed the sustained reduction in lung cancer mortality among those screened with LDCT over CXR. This persistent difference suggests that LDCT screening not only delays lung cancer deaths but also reduces mortality from lung cancer by detecting tumors at earlier stages [14].

Subsequent randomized controlled trials in other countries also showed a mortality reduction with LDCT screening. In 2003, the NELSON trial assessed the effectiveness of LDCT for lung cancer screening compared to no screening. The trial enrolled over 15,000 participants aged 50 to 74 years and demonstrated that LDCT screening resulted in a 24% reduction in lung cancer deaths over a 10-year follow-up period [5].

A 2005 study, known as the Multicentric Italian Lung Detection (MILD) trial, also examined the effectiveness of lung cancer screening with LDCT in over 4000 asymptomatic individuals aged 49 to 75 years with a history of smoking of at least 20-pack-years. The trial demonstrated a 39% reduction in lung cancer mortality over 10 years compared to no screening. The outcomes from the MILD trial further contributed to valuable evidence supporting the utilization of LDCT for effective lung cancer screening [15,16].

Table 1 presents a list of some of the largest lung cancer screening studies using LDCT, and a comparison of these has been performed previously in other reviews and meta-analyses [17,18].

### 2.2. Current Guidelines and Recommendations for Lung Cancer Screening

The evidence from clinical trials demonstrating an improved sensitivity with LDCT compared to CXR, particularly for smaller lesions, established LDCT as the recommended test for annual lung cancer screening [4,14,20,21]. The US Preventive Services Task Force (USPSTF), an independent panel of healthcare experts that establish evidence-based recommendations on clinical preventive services, issued a B grade recommendation in 2013, two years after results from the NLST were published. The recommendation called for annual lung cancer screening with LDCT in adults aged 55 to 80 years with a 30-pack-year smoking history who currently smoke or quit within the past 15 years [22]. The B grade signifies “at least fair evidence that [the service] improves important health outcomes and concludes that benefits outweigh harms” [22]. Since the passage of the Affordable Care Act of 2010, a B grade from the USPSTF requires most private plans to cover the test without cost-sharing to the beneficiary [22,23]. In 2021, the USPSTF lowered the eligibility age for lung cancer screening to 50 years and reduced the pack-year requirement to 20-pack-years [17]. This update was intended to improve equity in lung cancer screening by reducing disparities in access to screening among different racial and ethnic groups, particularly among Black Americans, in whom lung cancer development has been identified at younger ages with lower smoking intensity [24]. Studies have evaluated the impact of the modifications in the USPSTF lung cancer screening eligibility criteria between 2013 and 2021 within Black and White community-dwelling adults and found that the 2021 changes were associated with a reduction of existing eligibility disparities in lung cancer screening [25]. These findings emphasize the importance of factors beyond age and pack-years of smoking when establishing guidelines.

In 2023, the American Cancer Society (ACS) updated its lung cancer screening recommendations to match USPSTF, with the exception of the years since quit criteria, which was removed [26]. This update from previous guidelines underscores a concerted effort to streamline and improve access to LDCT screening for individuals who remain at elevated risk of lung cancer. The ACS recommendation highlights a shift in the lung cancer screening landscape toward an effort to expand access and catch more cancers early.

The USPSTF aims to update screening recommendations every 5 years using new research and data to support recommendations. The 2021 lung cancer screening recommendation highlighted several research gaps, emphasizing the necessity for implementation studies to promote health equity in screening uptake and additional evidence on biomarkers to identify individuals at higher risk for lung cancer. These areas were listed as research priorities for future recommendations [17].

Currently, both USPSTF and ACS guidelines recommend screening for patients aged 50 to 80, who currently or previously smoked with a 20-pack-year history [26,27]. The USPSTF includes the criteria that screening should be undertaken in patients who have quit within the last 15 years.

### 2.3. Factors Contributing to the Low Uptake of Lung Cancer Screening

Despite positive results from multiple randomized controlled trials and strong recommendations for lung cancer screening from USPSTF and leading cancer societies, screening utilization remains low. Some experts within the lung cancer community attribute the low screening uptake to the additional requirements around years since an individual quit smoking, as a history of smoking is often not recorded accurately in medical records or not recorded at all. Other factors that contribute, often in tandem, include limited or misinformed public knowledge on the harms of LDCT, difficulty accessing a facility with an LDCT scanner, financial costs from time off work coupled with structural barriers within the healthcare system and inadequate insurance coverage that make it difficult for providers and patients to navigate [28]. Additionally, the stigmatization and shame associated with smoking contribute to why those who smoke avoid disclosing their smoking history and status, making it difficult for providers to identify individuals eligible for screening.

Tobacco marketing campaigns have historically targeted marginalized and underserved communities [24], and it is these same communities that experience the highest incidence and mortality rates from the disease. Studies have identified disparities in the utilization of screening among eligible individuals and found that eligible non-Black patients were nearly three times more likely to undergo screening compared to eligible Black counterparts [29]. The impact of structural and systemic barriers on lung cancer screening becomes especially pronounced in southern states that lack Medicaid expansion, which amplifies existing disparities in healthcare access for mainly Black and Latino populations [30].

Low referral rates also remain a critical barrier. They are more prominent in marginalized communities, often stemming from healthcare providers’ lack of awareness and uncertainties regarding the perceived advantages of undergoing lung cancer screening. Providers have attributed low referral rates to errors in the electronic medical record notification system [31]. For instance, nearly one-third of providers surveyed reported never ordering LDCT scans for eligible patients despite 83% of providers acknowledging the USPSTF recommendation for annual LDCT screening. For those who have ordered LDCT for lung cancer screening, the majority report only doing so an average of 1.5 times per month [31].

Studies assessing primary care providers’ knowledge of lung cancer screening found that most did not know the full eligibility criteria [31,32]. A 2020 survey found that only 24% of providers correctly identified the eligibility criteria for screening, and when challenged with incorrect options, only 6% identified all the eligibility criteria correctly [31]. A 2023 study among primary care and pulmonary care clinicians found knowledge related to the correct modality for lung cancer screening to be low regardless of clinical specialty, with most endorsing CXR as the correct modality for lung cancer screening when assessed with hypothetical patient vignettes [33]. Nearly 30% of providers did not order LDCT scans for eligible patients, and 62% of providers stated no patients asked about lung cancer screening. Experts have frequently attributed the low uptake to the availability of various screening guidelines, each with slightly different recommendations for patient selection, screening frequency, and screening duration [31,32,33,34]. The inconsistency of guidelines, lack of comprehensive and accurate smoking history data, and the absence of validated electronic algorithms to assess relevant health factors impede successful screening programs [35].

Despite the many obstacles hindering lung cancer screening, individuals who previously or currently smoke are adherent to other recommended cancer screenings, indicating their commitment to health [36]. This suggests that low uptake of lung cancer screening can be improved when preventative services are offered to all eligible individuals. Improving uptake requires addressing the multifaceted barriers at the individual, provider, and system levels that disproportionately impact specific communities. Understanding barriers is crucial for healthcare professionals, policymakers, and researchers to design and implement effective strategies for increasing the uptake of lung cancer screening. By comprehensively addressing these factors, we can move toward a future where lung cancer stigma is reduced. This will empower providers to confidently engage in screening discussions, leading to early detection, better patient outcomes, and reduced disease burden.

## 3. Breakthroughs in Cancer Research

### 3.1. Advances in Liquid Biopsy for Early Detection

Nearly 40 years after extracellular strands of DNA, known as cell-free DNA (cfDNA), were discovered in human plasma, researchers found that cfDNA shed from cancer cells held unique genetic alterations from where the tumor originated [37,38]. Initially, researchers focused on identifying genetic mutations associated with lung cancer, utilizing circulating tumor DNA (ctDNA) as a minimally invasive approach to qualitative and quantitative molecular profiling of NSCLC. However, its utilization presented significant challenges and limitations that affected the sensitivity of the analysis, including minimal amounts of ctDNA in early-stage cancer [39,40,41]. As technology has advanced, the emphasis has shifted towards leveraging these biomarkers for early detection in the asymptomatic individual.

A wide range of approaches have been developed to detect cancer using cfDNA, including identification of somatic single nucleotide variants (SNVs), a task complicated by the extremely low tumor content. The mutational burden associated with clonal hematopoiesis further confounds early cancer detection [42,43]. Addressing challenges associated with cfDNA has centered on devising methods for sensitive and accurate detection of low-frequency mutations (<1%) in stage I cancers [44], examining its composition, and translating research discoveries into practical clinical applications. Numerous proof-of-concept investigations have delved into the blood-based detection of lung cancer [45,46], but initial attempts continued to exhibit limited test sensitivity for detecting stage I disease with performance based on participants outside of the screening-eligible population, further limiting the applicability to clinical practice [46].

Researchers from Johns Hopkins University conducted a comprehensive analysis of cfDNA fragmentation patterns. This analysis aimed to overcome known limitations with deep targeted sequencing for mutations or methylation signals that have poor performance in early-stage cancers through identification of aberrant fragmentation patterns [42,43]. The discovery resulted in a novel blood-based cfDNA fragmentation approach offering the most recent advancement for early cancer detection positioned to effectively overcome the known limitations by achieving enhanced early-stage sensitivity. The cfDNA fragmentation method involves extracting cfDNA from plasma, constructing whole-genome libraries, performing low-coverage whole-genome sequencing, and employing a machine learning algorithm, resulting in the ability to identify the presence of cancer DNA in stage I disease [42,43].

Applying low-coverage whole-genome sequencing to assess cfDNA fragmentation profiles across the entire genome has yielded encouraging results [42]. The approach successfully characterized the profiles of cfDNA fragment patterns in healthy individuals and in patients with cancer and found sensitivities of detection from 57% to 99% among seven cancer types at 98% specificity (AUC = 0.94) [42]. This discovery has unveiled variations in coverage and fragment size distribution of cfDNA throughout the genome due to differences in chromatin structure and chromosome composition in cancer cells. The DELFI (DNA Evaluation of Fragments for Early Interception) method employs machine learning analyses of complete genome cfDNA fragmentation profiles to identify cancer and the probable tissue source [47], a new approach for noninvasive cancer detection.

Many promising techniques have been developed to enhance liquid biopsy across the continuum of lung cancer care. Other reviews have detailed the potential of not only cfDNA, but also circulating tumor cells, proteins, metabolites, RNAs, and epigenetic markers like methylation to improve screening [48], diagnosis [49], and follow up of lung cancer [50]. Therefore, it is the purpose of this review to focus on the evolution of lung cancer screening as it currently stands and genomic technologies which are currently commercially available for clinical use for lung cancer screening.

### 3.2. Applications for DELFI in the Early Detection of Lung Cancer

The DELFI lung test was developed with the intention of seamlessly integrating into the screening pathway, with the ultimate goal of improving early detection. The first clinical validation trial sponsored by DELFI Diagnostics, Inc. (“DELFI Diagnostics”) was a multicenter prospective case-control study (DELFI-L101, NCT04825834) to develop and validate machine learning-based classifiers for cfDNA fragmentation [51]. DELFI-L101 was completed in 2023 and enrolled individuals at an elevated risk of developing lung cancer per the 2021 USPSTF guideline recommendations [51]. This study provided the necessary data to support the commercialization of the DELFI lung test (*pWGFrag-Lung*, FirstLook Lung) [51].

The DELFI lung test, commercially available as FirstLook Lung, is a validated next-generation sequencing assay of plasma cfDNA that analyzes the distribution of cfDNA fragment sizes to indicate the presence of possible lung cancer [51]. The FirstLook Lung test is intended to be used by a qualified healthcare provider and is not a replacement for computed tomography (CT) or LDCT of the chest. FirstLook Lung is validated for use in individuals aged 50 or older who either currently or previously smoked cigarettes and accumulated 20-pack-years or more of exposure [51].

In April 2022, a larger event-driven, prospective, observational study (CASCADE-LUNG or DELFI-L201, NCT05306288) was launched to advance data development and attain broad payer coverage. This study completed enrollment in 2023 and is underway to evaluate the sensitivity and specificity of the DELFI-based test as an in vitro diagnostic (IVD) for the detection of lung cancer among individuals eligible for routine lung cancer screening [52,53]. A third study was initiated in 2023 (FIRSTLung L301 or DELFI-L301, NCT06145750) and designed as a prospective cluster randomized trial to determine whether the availability of the FirstLook Lung test in primary care practices affects overall lung cancer screening rates in the screening-eligible population [54].

In September 2022, DELFI Diagnostics was selected as the liquid biopsy partner for 4-IN-THE-LUNG-RUN (4ITLR, NTR NL9710), a European lung cancer screening study evaluating the optimal screening frequency for individuals with a negative initial CT scan for lung cancer [53,55]. This large prospective research study is a collaborative effort between the Netherlands Cancer Institute (NKI) and the Institute for Diagnostic Accuracy (iDNA) [56], and DELFI Diagnostics will analyze samples from 9000 study participants to measure who is most likely to benefit from a CT scan.

Table 2 presents a summary of the clinical trials evaluating FirstLook Lung. Several other methods are currently in various stages of development or validation (e.g., clinical trials NCT05117840, NCT04968548, and NCT062458760). At the time of this review, FirstLook Lung is the only commercially available cfDNA blood-based test intended specifically for lung cancer screening in the US.

### 3.3. Accessible Screening in the Screening-Eligible Population

Cancer screening tests accessible in the office or at home are powerful tools for increasing screening participation and reducing disparities to improve cancer outcomes, particularly in underserved communities. Characteristics of an effective screening test should be convenient and accessible in the primary care setting. The test performance should be reliable and reproducible, with a high sensitivity rate to detect cancer signals in stage I disease with a high negative predictive value [57]. The value of an early detection test with strong performance metrics lies in its ability to identify cancers early where better treatment options exist, reduce anxiety and complications associated with more invasive or unnecessary procedures, and optimize healthcare resources appropriately.

The convenience of fecal immunochemical tests (FIT) and guaiac fecal occult blood tests (gFOBT), for instance, have played a critical role in improving overall completion rates for colorectal cancer screening (CRC) [58,59]. These non-invasive, at-home tests streamline the allocation of colonoscopy resources to those who are more likely to benefit, optimizing the utilization of healthcare resources and improving the overall efficiency of CRC programs [60]. These tests also reduce the population level risk of complications from colonoscopies.

Insights from CRC screening tests have demonstrated that offering a range of options, particularly non-invasive and home-based alternatives, can significantly increase screening participation rates [61]. When deliberating the efficacy of various CRC screening tests, a frequently cited maxim by USPSTF is, “The best screening test is the one that gets done” [62]. This underscores the importance of accessibility, optionality, and individual preferences in determining the success of screening initiatives.

Similar to CRC screening, the frequency of complications from downstream procedures from lung cancer screening can pose significant health and financial burdens. Identifying non-cancerous nodules leads to invasive lung biopsies that could otherwise have been ignored. Further evidence suggests that the real-world population may experience more frequent complications from invasive procedures, with one study identifying that complications classified as major occurred twice as often compared to the rate observed in the NLST population [63]. A blood-based point-of-care test designed to enhance the utilization of LDCT scans among individuals at elevated risk of lung cancer has the potential to address numerous deficiencies within the existing screening pathway. This includes prioritizing screening for patients most likely to benefit while simultaneously reducing harm from unnecessary procedures.

A blood-based lung cancer screening test—with ease of use, high test sensitivity, and high negative predictive value—brings an accessible approach to screening for lung cancer by appealing to individuals eligible for LDCT, but for any number of reasons, they have not received one. Reasons for nonadherence to lung cancer screening guidelines can include the distance required to travel to an imaging center, cost (or perceived cost) of the procedure, lack of education or awareness of screening, and patient nihilism. Following a shared decision making visit, lack of adherence to screening is associated with the additional time required to attend an imaging appointment, and lack of perceived contact for follow-up [64,65,66]. A blood-based test could alleviate these issues by providing a point-of-care solution. It also has the potential to optimize the benefit-to-harm ratio by identifying individuals who are most likely to derive benefits from LDCT screening while minimizing potential harm to those unlikely to benefit [48,67]. Results from such a test can provide a more informed screening decision for patients and their providers. Optimally, such a test should reduce the number needed to screen (NNS). A reduced NNS translates to a higher yield of positive findings on a subsequent screening LDCT, enabling providers to identify cases of concern while mitigating potential harms associated with unnecessary testing.

A new blood-based lung cancer screening test shows promise in increasing uptake among individuals who have not been screened. This advancement aims to improve screening effectiveness and decrease disease burden. Personalized interventions with a point-of-care blood test address specific barriers to screening, improving adherence and encouraging more of the eligible population to undergo annual screening.

## 4. Future Direction

Lung cancer screening faces a unique confluence of barriers despite robust data showing early detection with LDCT is efficacious. Imaging for lung cancer screening has significantly advanced early detection efforts; however, it has not been without limitations. Blood-based testing offers several advantages to the lung cancer screening pathway by addressing some of the limitations associated with imaging, such as concerns around excess radiation exposure, accessibility to an LDCT scanner, and travel time [12,31,68,69,70], considering blood-based tests can be drawn in clinical settings or in-home [71,72]. Routine testing familiarity is also a significant factor, with point-of-care blood tests often a part of regular primary care check-ups [71,72]. These factors contribute to patients’ preference for blood tests [73].

Additionally, LDCT uncovers non-cancerous nodules that often require multiple follow-up procedures for diagnosis, which is costly, time-consuming, anxiety-inducing, and invasive [20]. This detection of non-cancerous nodules is a frequently described deterrent for healthcare providers, represented by a reduced frequency of ordering LDCT scans [74]. Blood-based tests offer a promising solution to alleviate these concerns by delivering accurate true-positive detection results and offering personalized screening recommendations by providers to patients. These tests empower healthcare providers and patients to make informed decisions about ongoing screening while also reducing the demand for LDCT scans among individuals less likely to be diagnosed with lung cancer [36].

Increased accessibility can lead to higher participation rates in lung cancer screening programs and cater to a portion of the screening-eligible population that would otherwise not have elected to be screened. Such improvements can enhance screening and health outcomes among individuals from socioeconomically disadvantaged backgrounds. This is a population identified by the US National Cancer Institute (NCI), Surveillance, Epidemiology, and End Results (SEER), World Health Organization (WHO), and International Agency for Research on Cancer (IARC) that has historically lacked equal access to cancer screening, treatment, and clinical trial participation, which results in later stage diagnosis and worse patient outcomes [75,76].

With more elevated-risk individuals entering screening, greater public health and economic improvements could be realized. Lung cancer care was ranked third highest in 2020 annual expenditures by cancer type at nearly $24 billion [77]. Treatment costs for late-stage lung cancer in the initial year were nearly 2–3 times higher than those for early-stage treatment [78]. Additionally, mortality rates decreased for cancers with screening modalities [4,5]. This highlights the importance of focusing on early-stage lung cancer screening and detection.

## 5. Conclusions

The evolution of lung cancer screening has been a dynamic process driven by technological advancements, new research about the disease, and a growing emphasis on early detection. The advancements reflect a shift towards more accurate and efficient detection strategies. Chest radiography, once the primary tool for identifying lung abnormalities, is now typically reserved for diagnostic imaging in the US and is not recommended for lung cancer screening [13].

The persistent challenge of low lung cancer screening uptake remains a significant concern. This issue is influenced by various factors encompassing public awareness, perception, socioeconomic disparities, and systemic healthcare barriers. The substantial benefits associated with early detection are contingent upon collective endeavors to address these barriers and enhance screening program participation.

Healthcare providers play a crucial role in facilitating open and informed dialogues with patients regarding lung cancer screening, reducing the stigma associated with smoking, and enhancing shared decision-making discussions to effectively educate patients on the significance of early detection and preventive measures. Innovative technologies like noninvasive blood tests for lung cancer screening show significant promise, with several advancements currently in development and varying in progress.

While the path ahead presents challenges, it also offers a clear direction forward. A collective call to action is essential, rallying healthcare professionals, policymakers, and researchers to join this expansive endeavor. Policymakers wield considerable influence in addressing healthcare disparities, improving accessibility to screening facilities, and alleviating financial barriers through legislative and policy adjustments. These interventions have the potential to establish equitable access to screening services, ensuring that all individuals have the opportunity to benefit from early detection.

Ongoing blood-based biomarker research should prioritize standardizing protocols, ensuring clinical validation in the target population, and emphasizing clinical utility. Screening efforts should focus on achieving high sensitivity for early-stage detection and maintaining a robust negative predictive value to maximize the effectiveness of prompt and accurate lung cancer detection. Deploying comprehensive strategies, incorporating innovative fragmentomic technologies, and ensuring access can overcome the longstanding obstacles preventing progress. These collaborative endeavors aim to redefine the framework of lung cancer screening, ultimately reducing the impact of this disease, improving patient outcomes, and contributing to a shift in public health.

## Figures and Tables

**Table 1:** Clinical trials evaluating lung cancer screening.

Trial	Year Published	Location	Population
LSS	2005	United States	1660
DLCST	2009	Denmark	4104
NLST [4]	2011	US	53,454
ITALUNG	2013	Italy	1613
DANTE	2015	Italy	2472
LUSI	2015	Germany	4052
MILD	2016	Italy	2376
BRELT-1	2016	Brazil	790
UKLS [19]	2016	United Kingdom	4055
NELSON [5]	2020	Netherlands and Belgium	15,789
BRELT-2	2022	Brazil	3470

**Table 2:** Clinical trials evaluating *pWGFrag-Lung*.

Study	Design	Purpose	Inclusion Criteria	Enrollment	Trial Registration
L101	Case Control	Training/Validation	≥50 yo, current or former smoker, ≥20 pack years	2500	NCT04825834
L201	Cohort	Validation	≥50 yo, current or former smoker, ≥20 pack years	15,000	NCT05306288
4-ITLR	Cohort	Validation	60–79 yo, current or former smoker, ≥35 pack years	9000	NTR NL9710
L301	Randomized Cluster Trial	Evaluate Screening Uptake	Primary Care Practices	≤90 practices	NCT06145750

## Data Availability

No new data were created or analyzed in this study. Data sharing is not applicable to this article.
